# The use of optical coherence tomography angiography in comparing choriocapillaris recovery between two treatment strategies for multifocal choroiditis: a pilot clinical trial

**DOI:** 10.1186/s12348-022-00291-5

**Published:** 2022-03-11

**Authors:** Aniruddha Agarwal, Khushdeep Abhaypal, Kanika Aggarwal, Roel J. Erckens, Tos T. J. M. Berendschot, C. A. B. Webers, Mohit Dogra, Reema Bansal, Vishali Gupta

**Affiliations:** 1 Eye Institute, Cleveland Clinic Abu Dhabi, Al Maryah Island, Abu Dhabi, United Arab Emirates; 2 Advanced Eye Center, Post Graduate Institute of Medical Education and Research, Sector 12, Chandigarh, India; 3 Ahalia Eye Care, Delma St, Airport Road, Abu Dhabi, United Arab Emirates; 4 Maastricht University Medical Centre, University Eye Clinic Maastricht, Maastricht, The Netherlands

**Keywords:** Optical coherence tomography angiography, Multifocal choroiditis, Choriocapillaris, Flow deficit, Imaging, Uveitis

## Abstract

**Purpose:**

To compare differences in choriocapillaris flow deficit (CC FD) in multifocal choroiditis (MFC) between two treatment arms using optical coherence tomography angiography (OCTA).

**Methods:**

In this prospective randomized clinical trial, patients were randomized to either Group 1 which received standard tapering dose of oral corticosteroids, or Group 2 which received additional dexamethasone implant (or intravitreal methotrexate). The patients were followed-up until 12 weeks using OCTA and other imaging tools. CC FD and visual acuity between the two groups were compared at each visit.

**Results:**

Twenty-five subjects (17 males; 25 eyes) were studied (11 eyes in Group 1). There were no differences between the visual acuity or CC FD (1.12 versus 1.08 mm^2^; *p* = 0.86) at baseline between the groups. However, patients in Group 2 achieved better visual acuity (0.32 ± 0.23 versus 0.15 ± 0.11; *p* = 0.025) and CC FD (0.54 versus 0.15 mm^2^; *p* = 0.008) at 12 weeks.

**Conclusions:**

OCTA is a useful tool in monitoring the CC FD recovery after treatment in MFC. Patients receiving intravitreal corticosteroid/methotrexate in addition to systemic corticosteroid showed greater resolution of CC FD on OCTA compared to those receiving only oral corticosteroids.

## Introduction

Multifocal choroiditis is a form of posterior uveitis that is associated with inflammation involving the choriocapillaris, retinal pigment epithelium (RPE), and secondarily, the outer retina [1, 2]. These entities represent a manifestation of choriocapillaritis, suggesting that the primary site of inflammation is the choriocapillaris [1, 2]. A number of entities can cause multifocal lesions of choroiditis, including autoimmune white dot syndromes (such as multifocal choroiditis) [3], and tubercular serpiginous-like choroiditis (TB SLC) especially in endemic countries [4, 5]. The disease activity can be demonstrated by using various imaging modalities such as fundus autofluorescence (FAF), indocyanine green angiography (ICGA) and fluorescein angiography (FA) [1, 6, 7].

Optical coherence tomography angiography (OCTA) is new non-invasive tool that enables the visualization of the retinal and choroidal vessels in vivo without dye injection. Combined with the conventional depth-resolved structural imaging capabilities of OCT, OCTAs is a powerful tool to visualize and investigate the retinal and choroidal circulation [8, 9].

Recently, lesions of TB SLC have been characterized using OCTA [10, 11]. In the acute stage, findings of OCTA agree well with other imaging techniques such as ICGA and enhanced depth image optical coherence tomography (EDI-OCT) in determining choriocapillaris hypo-perfusion [10, 12, 13]. After treatment, the OCTA has the capability of showing reduction in choriocapillaris flow deficit (CC FD) areas on en face imaging, which also correlates well with ICGA [11]. Thus, OCTA has the potential to be used as a clinical endpoint in the management of subjects with macular choroiditis.

Thus far, there are no studies that have compared the resolution of CC FD (i.e., hypo-perfusion) on OCTA based on the therapeutic interventions in multifocal choroiditis. Currently, oral corticosteroids form the standard of care for treatment of multifocal choroiditis (with concomitant anti-tubercular therapy in tubercular etiologies) [4, 14, 15]. We hypothesize that initial treatment with adjunctive intravitreal therapy in subjects of choroiditis may result in better recovery of the CC FD on OCTA imaging. In this study, we aim to compare the difference in the recovery of the choriocapillaris between standard therapy consisting of oral corticosteroids, and a combination therapy consisting of both systemic and local intravitreal therapies.

## Materials and methods

In this prospective, randomized controlled, interventional study, patients were recruited from the Uveitis Clinic of Advanced Eye Centre, Department of Ophthalmology, Post Graduate Institute of Medical Education and Research (PGIMER), Chandigarh. Twenty-five subjects with multifocal choroiditis meeting the inclusion and exclusion criteria (detailed below) were recruited in the study. These subjects were randomized into two treatment arms: Group 1 and Group 2. Since this was a pilot clinical trial, no formal sample size calculations were performed for the study. The study was registered under the Clinical Trials Registry – India (CTRI; ref.: CTRI/2020/09/028056) prior to the conduct of the study. The study was conducted after approval from the Institute Ethics Committee (IEC) of PGIMER, Chandigarh, India. Written informed consent was obtained from all participating subjects, and the study adhered to the tenets of the Declaration of Helsinki.

### Study subjects

Patients were allotted in either Group 1 or 2 based on randomized treatment allocation by envelope method. In this method, the investigator was given randomly generated treatment allocations within sealed opaque envelopes. Once a patient had consented to enter the trial an envelope was opened, and the patient was then offered the allocated treatment regimen. Patients (between 19 and 60 years of age, either gender) with the following inclusion criteria were enrolled in the study:

- Subjects diagnosed with active multifocal lesions of choroiditis in at least one eye involving the posterior pole. Subjects with diagnosis of either autoimmune multifocal choroiditis or TB SLC were included in the study.
- The disease activity was confirmed on clinical examination, and imaging techniques such as OCT, fundus autofluorescence (FAF), FA and ICGA [6].
- Subjects who were treatment-naïve or those who developed active disease with ≤10 mg/day oral corticosteroids.

The exclusion criteria were as follows: eyes in which media clarity was obscured by the presence of cataract, vitritis or any other such coexistent pathology that did not allow the acquisition of good images; subjects with active lesions outside the posterior pole that were not amenable to fundus imaging; subjects on treatment with long-term systemic immunosuppressive therapies, or those who have received intravitreal treatments in the past. In addition, subjects who have undergone pars plana vitrectomy (at any time point), cataract surgery (within the last 3 months), or glaucoma surgery (within the last 3 months) were excluded; subjects who were pregnant or plan to become pregnant during the course of therapy; subjects with placoid choroiditis characterized by large, plaque-like lesions involving large areas of the retinochoroid; subjects with concomitant ocular diseases such as diabetic retinopathy, optic neuropathy, or retinal degeneration, and patients who did not agree to come for follow-up visits.

### Study procedures

A detailed history was taken for all recruited patients i.e., presenting ocular complaints with their respective duration, any history suggestive of other co-existing ophthalmological diseases, previous treatment if taken, any known systemic illness and any known drug allergy. Best-corrected visual acuity (BCVA) of the patients was recorded using the Snellen’s visual acuity chart at baseline and at all follow up visits. BCVA was converted to LogMAR units for statistical analysis. Intraocular pressure (IOP) measurement by non-contact tonometer was done for all patients at baseline and at all follow up visits. Anterior segment examination using slit lamp was done in all patients at baseline and at all follow up visits. Posterior segment findings were noted by slit lamp biomicroscopy with + 90 D diopter lens. Peripheral fundus examination was done using the indirect ophthalmoscope with a + 20-diopter lens at baseline and at all follow-up visits.

Color fundus photographs were captured using Carl Zeiss Visupac FF450 fundus camera setting central 50 degrees (Carl Zeiss Meditec, Dublin, CA, USA) at baseline and all follow-up visits. OCTA (DRI TopCon Triton®, TopCon Inc., Tokyo, Japan) (3 × 3 mm scan) was performed at baseline and all follow-up visits. The OCTA images were analyzed for the choriocapillaris alterations at the site of lesions. Two independent masked graders (uveitis specialists with experience in imaging research: A.A. and K.A.) performed the image analysis on OCTA (blinded to the treatment groups and the time interval). The CC FD areas were measured manually in mm^2^ using a third-party software (ImageJ, National Institutes of Health, Bethesda, USA) [11]. Briefly, the area of CC FD was manually mapped by the two graders on ImageJ (in mm^2^) after standardizing the images and setting the scale, and the average of the two graders’ readings were used for the analysis. Swept-source (SS)-OCT (DRI TopCon Triton®, TopCon Inc., Tokyo, Japan) was performed at baseline and all follow-up visits. Combined FA and ICGA (Spectralis®, Heidelberg Engineering, Heidelberg, Germany was performed at baseline and 12 weeks. The two graders also performed the manual area measurements of the hypofluorescent areas on ICGA in the central 3 × 3 mm (identified after drawing a central 3 × 3 mm square) using the area measurement tool on Heyex Eye Explorer (version 1.10.4.0).

### Study arms, visits, and treatments

#### Group 1

Group 1 received standard treatment for multifocal choroiditis which consisted of oral corticosteroids (oral prednisolone 1 mg/kg/day) initiated at baseline and continued for a total of 8 weeks with tapering doses (5 mg/kg/week taper). If the subjects tested positive for TB (in subjects with tubercular serpiginous-like choroiditis), additional anti-tubercular therapy was given as per the following protocol: (induction phase) isoniazid (5 mg/kg/day), rifampicin (450 mg/day if body weight is≤50 kg and 600 mg/day if body weight is > 50 kg), pyrazinamide (25 to 30 mg/kg/day) and ethambutol (15 mg/kg/day). After 2 months, only isoniazid and rifampicin were continued for additional 7 months (maintenance phase). Liver function tests and hemograms were monitored every 6 weeks.

#### Group 2

In addition to standard treatment Group 2 patients received intravitreal dexamethasone implant (0.7 mg) injection at baseline. If the use of intravitreal corticosteroid was contraindicated (due to cataract, or steroid responsiveness), then the subjects received weekly injection of intravitreal methotrexate (400 μg/0.1 mL) every week for the first 4 weeks of treatment.

#### Study duration and follow-up

The duration of the study was 12 weeks. All the subjects were examined at baseline, 1 week, 2 weeks, 4 weeks, and 12 weeks.

### Outcome measures and statistical analysis

The main outcome measure of the study was the mean difference in the CC FD areas between the two treatment arms (indicative of choriocapillaris recovery). Other outcome measures included change in the BCVA between the two treatment arms, and the occurrence of adverse events in either arm.

The statistical analysis was done with the help of SPSS© version 26 for Windows (IBM Inc., Chicago, IL, USA). Data entries were performed in pre-designed forms and excel sheets using Microsoft Excel 2016© for Windows. All the measurable data was checked for their normality using Kolmogorov–Smirnov test within each group. The data was presented with descriptive statistics with mean ± standard deviation along with their range. To observe the treatment effect within each group, paired t test was applied to compare the differences in the CC FD areas before and after treatment. To see whether the treatment effect was equal between groups, Students t test was applied to compare the differences in the CC FD areas. Other parameters that were compared between the two groups included BCVA improvements at 12 weeks, changes in IOP, and adverse effects between the two groups. A *p* value < 0.05 was considered statistically significant.

## Results

In the study, twenty-five subjects (17 males; 25 eyes) with TB/SLC or MFC met the inclusion criteria and were evaluated. Thirteen eyes were diagnosed with TB SLC and 12 were diagnosed with MFC. Group 1 included 11 subjects (11 eyes) and group 2 included 14 subjects (14 eyes). The demographic and clinical details of the subjects, including their mean age, BCVA, IOP and diagnosis have been summarized in Table 1.

**Table 1:** Comparison of baseline demographic and clinical parameters between the two groups

	Group 1 (***n*** = 11 eyes)	Group 2 (***n*** = 14 eyes)	***P*** value
Age (years ± SD)	30.4 ± 8.3	32.4 ± 11.5	0.62
Gender (n)			0.20
Male	6	11	
Female	5	3	
Diagnosis (n)			0.82
TB SLC	6	7	
MFC	5	7	
Laterality (n)			0.89
Right eye	6	8	
Left eye	5	6	
Treatments (n)			
Intravitreal DEX	–	12	–
Intravitreal MTX	–	2	–
ATT	6	7	–
Oral corticosteroids	11	14	–
Initial BCVA (LogMAR units)	0.41 ± 0.25	0.38 ± 0.23	0.85
Final BCVA (LogMAR units)	0.32 ± 0.23	0.15 ± 0.11	0.025
(*p* value)*	(0.01)	(< 0.001)	
Initial IOP (mm Hg)	13.6 ± 1.7	13.7 ± 2.2	0.92
Final IOP (mm Hg)	14.1 ± 2.1	14.4 ± 3.8	0.82
(*p* value)*	(0.51)	(0.58)	

*p value has been calculated compared to baseline

*ATT* Anti-tubercular therapy, *BCVA* Best-corrected visual acuity, *DEX* Dexamethasone implant, *IOP* Intraocular pressure, *MFC* Multifocal choroiditis, *MTX* Methotrexate, *TB SLC* Tubercular serpiginous-like choroiditis

The group 1 (standard oral therapy) were given oral corticosteroid therapy in a tapering dose (as described in the methods). All eyes with TB SLC received concomitant ATT (in either group). In the combination group 2 (standard oral + intravitreal therapy), 2 eyes received intravitreal methotrexate due to history of corticosteroid responsiveness (one eye received 3 injections and the other received 2), whereas 12 eyes received intravitreal dexamethasone implant (single injection at baseline).

**Fig. 1:**
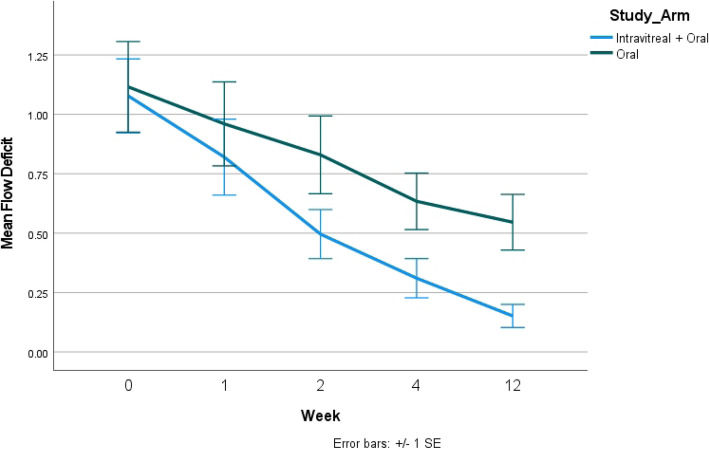
Fig. 1 compares the choriocapillaris flow deficit (CC FD) between the two study arms measured on optical coherence tomography angiography (OCTA). The CC FD reduced significantly at 12 weeks in the combination arm (intravitreal dexamethasone implant and oral corticosteroids) compared to oral corticosteroids alone

Analysis of OCTA in groups 1 and 2 showed a baseline mean CC FD of 1.12 mm^2^ and 1.08 mm^2^, respectively (*p* = 0.86). During follow-up, the CC FD improved in all eyes in both the groups (statistically significant at all time points compared to baseline). In comparing group 1 and 2, the recovery of choriocapillaris (measured in terms of decrease in CC FD) was significantly higher in eyes belonging to group 2 (Fig. 1). Table 2 provides a summary of the changes in OCTA CC FD from baseline through week 12 in both the groups.

**Table 2:** Comparison of optical coherence tomography angiography (OCTA) derived choriocapillaris flow deficit (CC FD) in mm^2^ in both the groups

	Group 1 (***n*** = 11 eyes)	Group 2 (***n*** = 14 eyes)	***P*** value
Baseline	1.12 ± 0.63	1.08 ± 0.58	0.86
1 week	0.96 ± 0.59	0.82 ± 0.60	0.56
***P*** value*	**< 0.001**	**< 0.001**	
2 weeks	0.83 ± 0.54	0.50 ± 0.39	0.086
***P*** value*	**< 0.001**	**< 0.001**	
4 weeks	0.63 ± 0.39	0.31 ± 0.31	**0.030**
***P*** value*	**< 0.001**	**< 0.001**	
12 weeks	0.54 ± 0.39	0.15 ± 0.18	**0.008**
***P*** value*	**< 0.001**	**< 0.001**	

*p value has been calculated compared to baseline

**Fig. 2:**
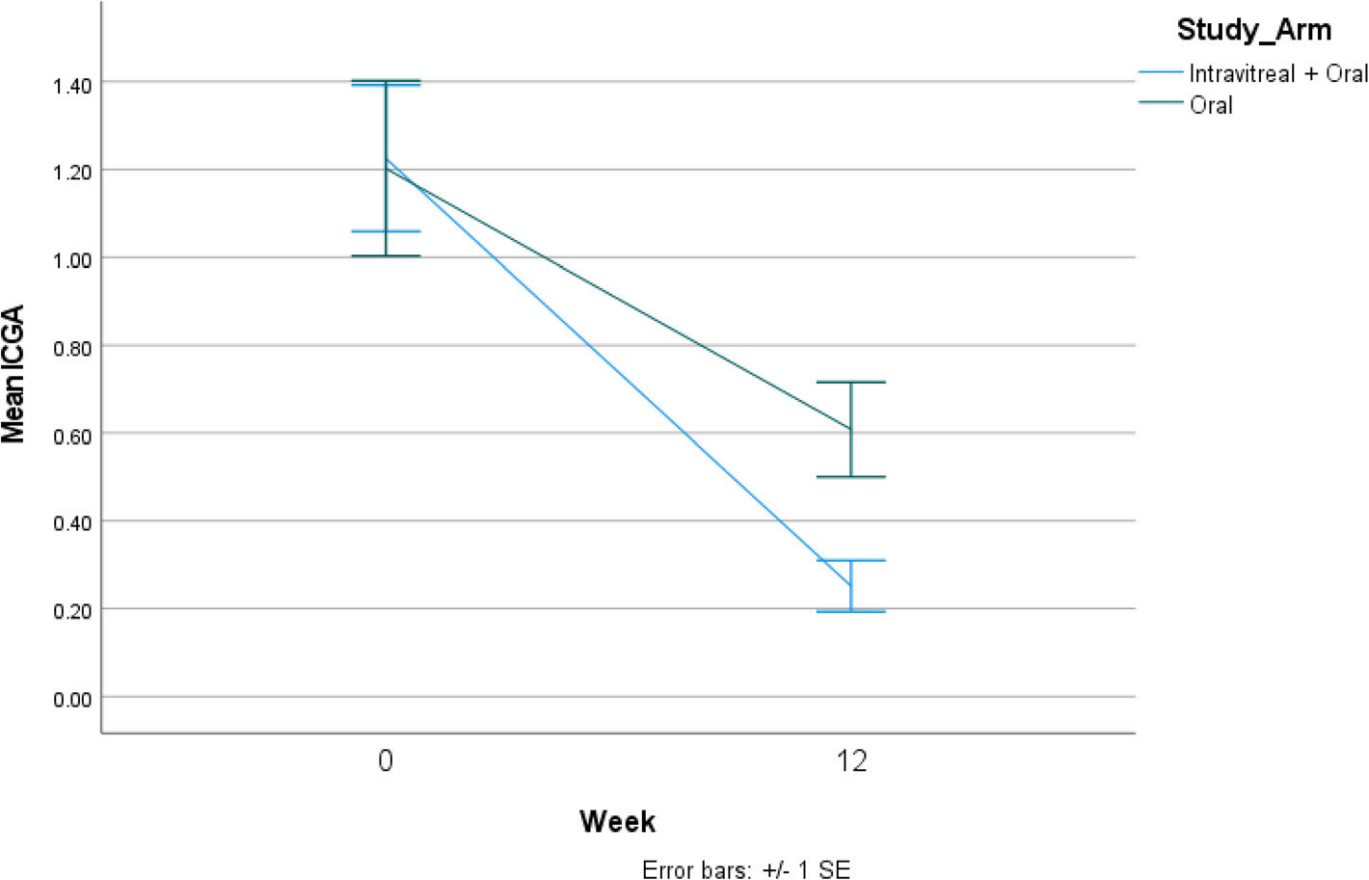
Fig. 2 compares the mean area of the choriocapillaris flow deficit on indocyanine green angiography (ICGA) imaging. Eyes in the combination arm (intravitreal dexamethasone implant and oral corticosteroids) had lesser flow deficit areas compared to eyes treated with oral corticosteroids alone at 12 weeks

On ICGA, the mean area of flow deficit at baseline was 1.20 mm^2^ in group 1 and 1.22 mm^2^ in group 2 (*p* = 0.93). At 12 weeks, the mean area of flow deficit was 0.61 mm^2^ in group 1, and 0.25 mm^2^ in group 2 (*p* = 0.005) (Table 3) (Fig. 2).

**Table 3:** Calculation of choriocapillaris flow deficit areas (in mm^2^) on indocyanine green angiography (ICGA) in both the groups

	Group 1 (***n*** = 11 eyes)	Group 2 (***n*** = 14 eyes)	***P*** value
Baseline	1.20 ± 0.66	1.22 ± 0.62	0.93
12 weeks	0.61 ± 0.36	0.25 ± 0.22	**0.005**
***P*** value*	**< 0.001**	**< 0.001**

**Fig. 3:**
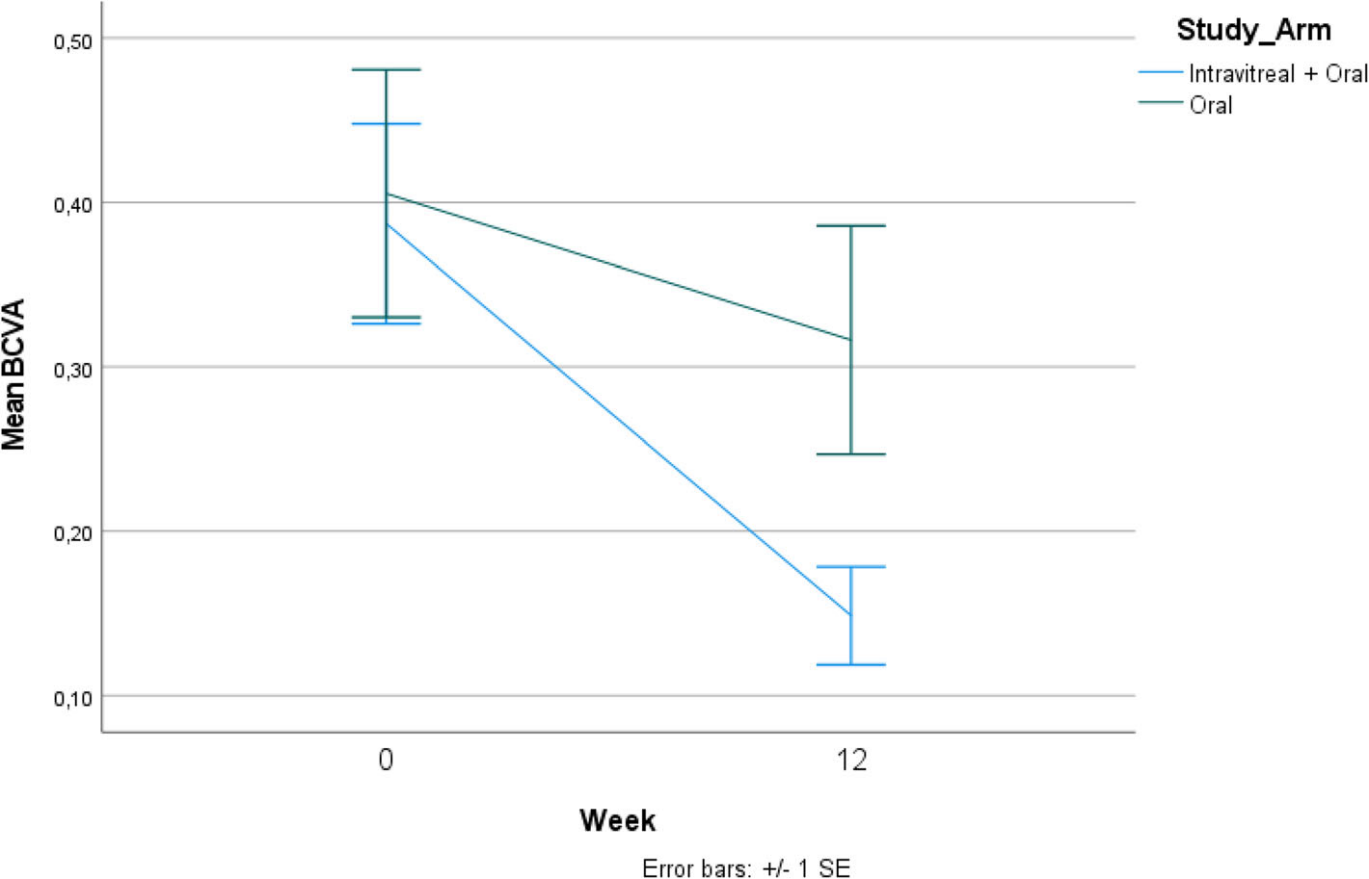
Fig. 3 shows the improvement in best-corrected visual acuity (BCVA) between the two treatment arms. Eyes in the combination arm (intravitreal dexamethasone implant and oral corticosteroids) had significantly better BCVA at 12 weeks compared to eyes receiving oral corticosteroids alone

The BCVA improved from 0.41 to 0.32 LogMAR units in group 1 (*p* = 0.014), whereas it improved from 0.39 to 0.15 LogMAR units in group 2 (*p* < 0.001). The BCVA was also significantly better in eyes belonging to group 2 at 12 weeks (*p* = 0.025) (Fig. 3).

**Fig. 4:**
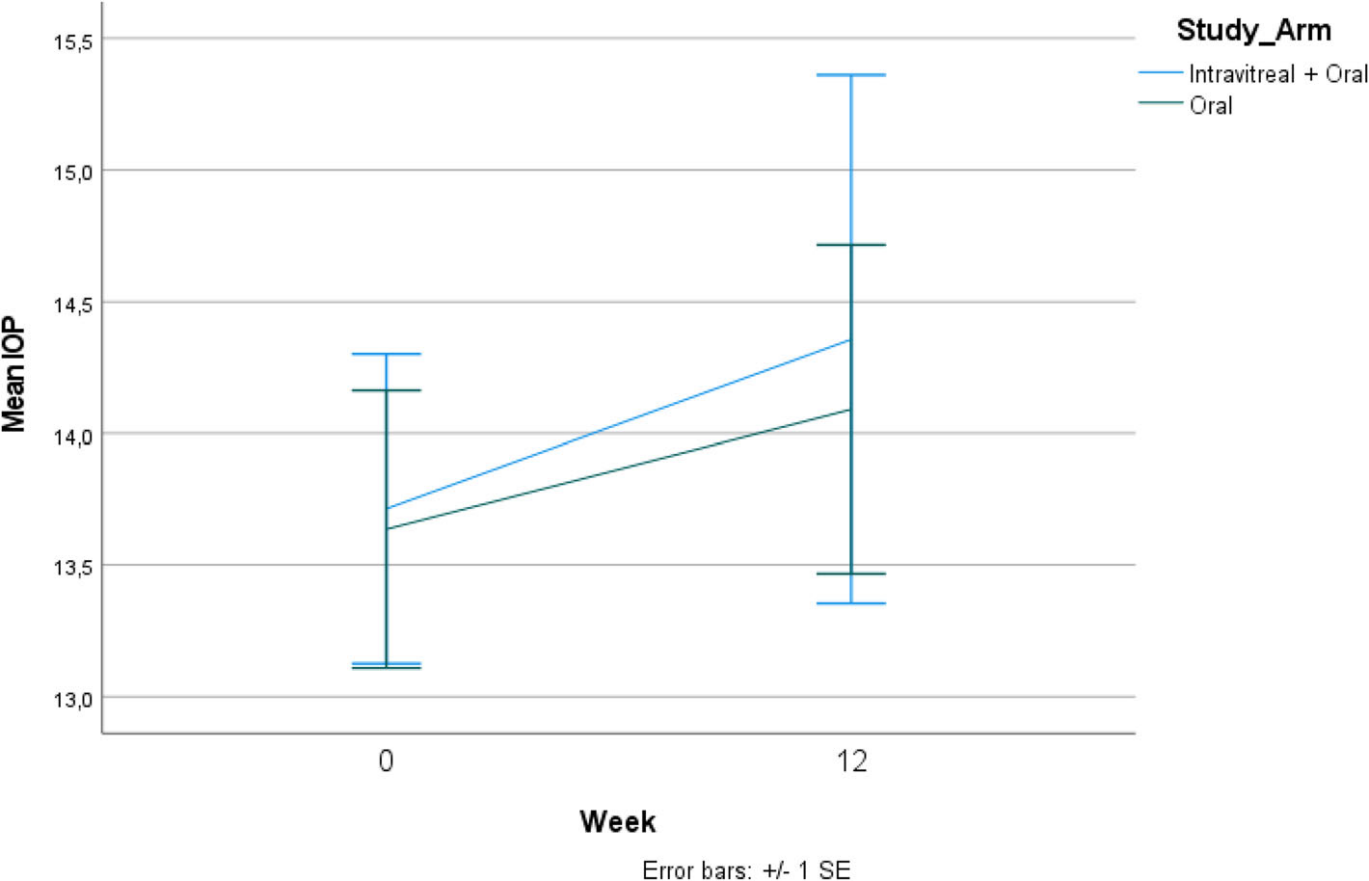
The figure shows change in the mean intraocular pressure (IOP) in the two treatment arms. While the IOP increased at 12 weeks in both the treatment arms compared to baseline, there were no statistically significant changes from baseline, or between the two treatment arms

The mean IOP increased from 13.6 to 14.1 mmHg in group 1 (*p* = 0.518), whereas it increased from 13.7 to 14.4 mmHg (*p* = 0.58) in group 2 (Table 1). Two eyes in group 2 were given topical dorzolamide 2% for 12 weeks (single agent) since the IOP was recorded > 21 mmHg (maximum of 25 mmHg) at week 2 in both eyes (Fig. 4).

None of the eyes in either group developed worsening of inflammation, increase in choroiditis lesions, development of new choroiditis lesions, or other adverse events such as endophthalmitis. All the eyes in both groups showed healing of the choroiditis lesions at the end of 12 weeks. In eyes requiring ATT, the therapy was continued beyond the period of the study i.e., 12 weeks.

## Discussion

In a previously published study by our group wherein patients with TB SLC were serially followed up using OCTA and ICGA imaging, it was observed that OCTA functions well in assessing the recovery of CC FD after initiation of treatment. The improvements in choriocapillaris during follow-up was also seen on ICGA at 3 months. An important highlight of the study was that lesions with an area greater than 0.1 mm^2^ at baseline showed healing but with significant choriocapillaris atrophy. Lesions smaller than 0.1mm^2^ in area showed near-complete resolution with minimal residual alterations in the choriocapillaris on ICGA [11]. Thus, the bigger the lesion area, the greater chances of choriocapillaris atrophy. Since then, there are very few studies published that demonstrate quantitative improvements in CC FD in posterior uveitis, specifically TB SLC and MFC [16–19].

We performed this study to assess whether adjunctive treatment with intravitreal corticosteroids/methotrexate can aid in the greater recovery of CC FD in cases with center-involving TB SLC and MFC. Atrophy of choriocapillaris due to inflammation involving the RPE, outer retina and choriocapillaris layer is associated with permanent visual loss and scarring [4]. Therefore, the aim of the treatment is to prevent as much central photoreceptor loss as possible. In this study, we observed improvements in CC FD in all eyes at week 12 compared to baseline, however, eyes receiving additional intravitreal dexamethasone implant/methotrexate performed significantly better with greater resolution of CC FD on OCTA and flow deficit areas on ICGA.

The application of quantitative metrics in posterior uveitis may be valuable to assess the effect of therapeutic interventions. Using easily available third-party software, it is possible to measure the areas of CC FD on OCTA [11, 18, 19]. In addition, the measurement of the hypofluorescent area on ICGA has been made possible by the tools available on the Heidelberg Eye Explorer, and similar in-built software on other commercially available devices. Studies have previously shown the agreement between OCTA and ICGA in terms of choriocapillaris flow measurements [11, 19]. Serial quantitative metrics provide an objective assessment of the choriocapillaris recovery, and thus serves as an endpoint for therapeutic interventions. In our study, the recovery of choriocapillaris after intravitreal injections was significantly better than the standard arm receiving only oral corticosteroid therapy at all follow-up visits.

The resolution of CC FD on OCTA was accompanied by significant improvements in BCVA in both the groups. While the BCVA improved from baseline in both groups of patients receiving treatment, the group receiving adjunctive intravitreal therapy had significantly higher visual outcomes even though there was no significant difference in the BCVA at baseline between the two groups. This suggests that addition of adjunctive intravitreal therapy at baseline may not only translate into better anatomical outcomes on OCTA in terms of CC FD, but also potentially better visual results at 12 weeks.

In patients with TB SLC and MFC, the safety and efficacy of intravitreal therapy with dexamethasone implant and methotrexate has been published previously [20–23]. The advantages of intravitreal corticosteroid/methotrexate injection include high local delivery of anti-inflammatory therapy that can promptly act to reduce the inflammation, avoiding systemic side-effects of prolonged oral therapy. Studies have shown that intravitreal injection of these agents are not associated with worsening of choroiditis in any patient and help in the healing of the lesions with minimal adverse events [20–23]. Since steroid-responsiveness is a cause of concern in patients receiving dexamethasone implant, we used intravitreal methotrexate in patients who had history of high IOP after corticosteroid use. None of the subjects in our cohort had uncontrollable rise in IOP, and 2 subjects required a short course of single topical anti-glaucoma medication for IOP rise.

Our study is a pilot clinical trial which is mainly aimed at evaluating the role of OCTA in quantifying lesions of choroiditis after initiation of treatment. Thus, we do not propose use of intravitreal corticosteroids/methotrexate in every case of macular choroiditis. There are several challenges in the management of choroiditis due to ocular TB, as well as MFC and these patients require individualized therapy to tackle persistence of lesions, development of new lesions, and other aspects such as drug resistance [14, 24]. However, our study does demonstrate that adjunctive intravitreal agents may have an role in improving the visual outcomes of these patients, and such therapies may be increasingly employed for our future patients.

Our study has several limitations. We had a modest sample size of patients. However, we included patients with strict inclusion criteria such as treatment-naïve macular choroiditis with no prior intraocular treatment, and clear media for serial OCTA. In addition, we did not have automated measurements of CC FD in our study. With improvements in technology and application of quantitative algorithms, automated quantification is being increasingly employed [17, 18, 25]. In the future, such automated measurements may be applied to OCTA imaging of lesions of choroiditis as well. Since patients in group 2 received either dexamethasone implant or methotrexate therapy, it may be argued that group 2 included patients receiving two different drugs. However, the aim of the study was not to compare the treatment strategies themselves, but the utility of imaging modality (i.e., OCTA) for the follow-up assessment of these patients.

In conclusion, OCTA is not only useful in assessing areas of CC FD in eyes with choroiditis, but it can be used as a tool to monitor recovery of the choriocapillaris and assess the efficacy of systemic and intravitreal anti-inflammatory therapies. In patients with TB SLC and MFC who receive adjunctive intravitreal corticosteroid/methotrexate therapy in addition to systemic corticosteroids, greater decrease in the CC FD area can be visualized on OCTA, and this may translate into superior visual outcomes due to decreased choriocapillaris atrophy.

## Data Availability

The data related to the study will be available upon reasonable request from the corresponding author.
